# Detection of *Mycobacterium ulcerans* by the Loop Mediated Isothermal Amplification Method

**DOI:** 10.1371/journal.pntd.0001590

**Published:** 2012-04-03

**Authors:** Anthony Ablordey, Diana Ackon Amissah, Isaac Frimpong Aboagye, Ben Hatano, Toshio Yamazaki, Tetsutaro Sata, Koichi Ishikawa, Harutaka Katano

**Affiliations:** 1 Department of Bacteriology, Noguchi Memorial Institute for Medical Research, University of Ghana, Legon, Ghana; 2 Department of Animal Science and Conservation Biology, University of Ghana, Legon, Ghana; 3 Department of Pathology, National Institute of Infectious Diseases, Tokyo, Japan; 4 Division of Biosafety Control and Research, National Institute of Infectious Diseases, Tokyo, Japan; 5 AIDS Research Center, National Institute of Infectious Diseases, Tokyo, Japan; Fondation raoul Follereau, France

## Abstract

**Background:**

Buruli ulcer (BU) caused by *Mycobacterium ulcerans* (*M. ulcerans*) has emerged as an important public health problem in several rural communities in sub-Saharan Africa. Early diagnosis and prompt treatment are important in preventing disfiguring complications associated with late stages of the disease progression. Presently there is no simple and rapid test that is appropriate for early diagnosis and use in the low-resource settings where *M. ulcerans* is most prevalent.

**Methodology:**

We compared conventional and pocket warmer loop mediated isothermal amplification (LAMP) methods (using a heat block and a pocket warmer respectively as heat source for amplification reaction) for the detection of *M. ulcerans* in clinical specimens. The effect of purified and crude DNA preparations on the detection rate of the LAMP assays were also investigated and compared with that of IS*2404* PCR, a reference assay for the detection of *M. ulcerans*. Thirty clinical specimens from suspected BU cases were examined by LAMP and IS*2404* PCR.

**Principal Findings:**

The lower detection limit of both LAMP methods at 60°C was 300 copies of IS*2404* and 30 copies of IS*2404* for the conventional LAMP at 65°C. When purified DNA extracts were used, both the conventional LAMP and IS*2404* PCR concordantly detected 21 positive cases, while the pocket warmer LAMP detected 19 cases. Nine of 30 samples were positive by both the LAMP assays as well as IS*2404* PCR when crude extracts of clinical specimens were used.

**Conclusion/Significance:**

The LAMP method can be used as a simple and rapid test for the detection of *M. ulcerans* in clinical specimens. However, obtaining purified DNA, as well as generating isothermal conditions, remains a major challenge for the use of the LAMP method under field conditions. With further improvement in DNA extraction and amplification conditions, the pwLAMP could be used as a point of care diagnostic test for BU

## Author Summary

In order to develop a simple and rapid test that can be used to diagnose Buruli ulcer under field conditions, we modified the conventional LAMP assay by using a disposable pocket warmer as a heating device for generating a constant temperature for the test reaction and employed the use of crude sample preparations consisting of boiled and unboiled extracts of the clinical specimen instead of using purified DNA as the diagnostic specimen. Thirty clinical specimens from suspected Buruli ulcer patients were investigated by the modified LAMP (or pocket warmer LAMP) and the conventional LAMP, as well as IS*2404* PCR, a reference method for the detection of *Mycobacterium ulcerans*. There was no significant difference in the detection rate (63–70%) in all of the methods when purified samples were used for the tests. On the other hand the use of crude specimen preparation resulted in a drop in detection rate (30–40%). This study demonstrates that the LAMP test can be used for rapid detection of *M. ulcerans* when purified DNA preparations are used. With further improvements in the sample reaction, as well as in specimen purification, the pocket warmer LAMP may provide a simple and rapid diagnostic test for Buruli ulcer.

## Introduction

Buruli ulcer (BU) caused by *Mycobacterium ulcerans* (*M. ulcerans*) is a necrotizing skin disease endemic mostly in rural wetland of tropical countries of Africa, America, Asia and Australia. The disease also occurs in non-tropical areas of Australia, China and Japan. Globally, BU has been reported in over 30 countries [1]–[3]. The burden of BU is however most severe in sub Saharan Africa where the true incidence of the disease is difficult to determine as a result of poor surveillance measures and case confirmation [2]. Available data however reveals an increase in BU incidence over the last several years in the west African countries of Ivory Coast, Ghana and Benin. In these countries BU has replaced leprosy as the second most prevalent mycobacterial disease [1], [3]–[5].

BU begins as painless nodule, papule, plaque or edema that evolves into characteristic ulcers with undermined edges. If untreated, extensive ulceration (that can cover 15% of the body), scarring and contractures may cause serious functional disabilities in patients [5]–[7].

Unfortunately most patients seek treatment late and present with large ulcers [8]–[10]. Previously treatment of such lesions involved surgical removal of all the affected tissue and part of the surrounding tissues, eventually followed by skin grafting [9]–[12].

In 2004 antimycobacterial treatment alone (if necessary in combination with surgery) was introduced and has since been considered as the treatment of choice for BU [6], [13]–[16]. Laboratory confirmation of clinically suspected BU cases has therefore become crucial for the clinical management of BU [17].

Four laboratory tests are recommended for the diagnosis of BU. These include microscopic examination, culture, IS*2404* PCR and histopathological analysis. Microscopic examination detects 29%–78% of clinically suspected BU cases and is currently the only rapid and affordable test available for BU diagnosis in many endemic areas. The detection rate of culture is between 34%–79% and takes an average of 9–12 weeks to yield positive results. Culture therefore cannot be used for rapid laboratory confirmation of BU. Histopathological analysis is reported to detect 30% additional cases than other confirmatory tests, however this technique is restricted to external reference laboratories and are unavailable in peripheral health centres or district or regional hospitals. IS*2404* PCR has close to 96% sensitivity and is considered the method of choice for laboratory confirmation of BU [16]–[25].

The WHO recommends that at least 50% of cases must be confirmed by IS*2404* PCR before commencement of antibiotic therapy [22]–[25].

However technical difficulties (eg, cold chain requirement, stable power supply and qualified laboratory staff) limit the use of this diagnostic test in BU endemic areas.

A dry reagent PCR consisting of lyophilized PCR mix which is reconstituted with water for testing DNA was developed to simplify BU diagnosis by PCR [26] but this method also requires the use of a thermocycler, electrophoresis and gel imaging equipment and therefore similarly makes the use of this diagnostic test for BU diagnosis in endemic areas unlikely.

The Loop Mediated Isothermal Amplification (LAMP) is a novel nucleic acid amplification method for molecular detection and identification [27]. The principle of LAMP is autocycling strand displacement DNA synthesis in the presence of Bst DNA polymerase with high strand displacement activity under isothermal conditions between 60–65°C within 60 minutes [28]. The assay is highly specific due to the recognition of target DNA by 4 to 6 independent sequences and the amplification efficiency of LAMP is equivalent to that of PCR based methods ([27], [29], [30]).

The LAMP reaction enables easy identification of positive tests due to the accumulation of high amounts of amplification products in the reaction tubes. Further improvement in visual identification can be realized through the addition of intercalating dyes such as SYBR green or hydroxynapthtol blue to reaction tubes [31]. This therefore precludes the need for post amplification analysis and hence reduces cost and labour. LAMP has also been shown to be less affected by a number of inhibitors of conventional PCR [32]. Additionally the closed tube format of this assay reduces problem of carry over contamination which is likely in less controlled environments [33]. With all of these characteristics LAMP of DNA has emerged as a powerful tool to facilitate point of care diagnostic test [31].

In order to develop a field applicable technique that offers high detection sensitivity and specificity for the diagnosis of BU, we explored the use of the pocket warmer LAMP (pwLAMP) technique, a DNA amplification method using isothermal conditions (60°C) provided by a disposable pocket warmer [34].

## Materials and Methods

### Ethics statement

Ethical approval for analysing patients' specimens was obtained from the ethical review board of the Noguchi Memorial Institute for Medical Research. Specimens used were anonymously taken from an already existing collection of patients' specimens processed for diagnosis of BU from Agogo Presbyterian Hospital in Ghana.

### DNA from clinical specimens

Thirty clinical specimens consisting of 20 swabs and 10 fine needle aspirates taken respectively from ulcers and pre-ulcerative lesions of suspected BU patients were used in this study. The fine needle aspirate specimens were kept in 1 ml phosphate buffered saline (PBS) and swabs were stored dry in sterile tubes. Each swab was transferred into a tube containing 2 ml milli-Q purified water (Millipore Corporation, Billerica, MA) and gently vortexed for 5 sec and then removed. Portions 250 µl of the sample suspensions were transferred to separate new sterile eppendorf tubes containing 250 µl of lysis buffer (1.6 M GuHCl, 60 mM Tris pH 7.4, 1% Triton X-100, 60 mM EDTA, Tween-20 10%), 50 µl proteinase-K and 250 µl glass beads.

The mixtures were incubated horizontally in a shaker (200 rpm) at 60°C overnight. To capture the DNA, 40 µl of diatomaceous earth solution (10 g diatomaceous earth obtained from Sigma Aldrich Chemi GmbH in 50 ml of H_2_O containing 500 µl of 37% (wt/vol) HCl) was added to the suspensions and incubated at 37°C with shaking (200 rpm) for 60 min. The mixtures were centrifuged at 14,000 rpm for 10 sec and the resulting pellets were washed twice with 900 µl of 70% ethanol (2–8°C) followed by 900 µl of acetone. The pellets were dried at 50°C for 20 min and resuspended in 100 µl milli Q purified water and centrifuged at 14,000 rpm for 10 sec. The purified DNA was used as templates for both IS*2404* PCR and LAMP assays to detect *M. ulcerans*.

To investigate the performance of the LAMP assay on crude DNA preparations, we obtained 2 types of DNA extracts for each clinical specimen. One crude extract consisted of 250 µl suspensions of the specimen boiled for 10 min followed by centrifugation at 14,000 rpm for 5 min (boiled extract). The other crude extract used was a 250 µl suspension of the unboiled specimen.

### Detection limit of LAMP

Ten *M. ulcerans* strains grown on LJ slants were harvested and DNA was extracted as previously described [35]. Serial dilutions of purified *M. ulcerans* DNA containing 300,000, 30,000, 300, 30 and 3 copies of IS*2404* element per 5 µl were prepared. The number of copies of the insertion sequence element was determined based on the genome size of 5,806 kb and presence of an average number of 207 copies of IS*2404*. This was used to determine the detection limit of the LAMP assays.

### PCR for IS*2404*

PCR targeting IS*2404* was performed as described previously [21]. The first and second round PCRs used primers pGp1: 5′-AGGGCAGCGCGGTGATACGG-3′and pGp2: 5′- CAGTGGATTGGTGCCGATCGAG-3′ and pGp3: 5′-GGCGCAGATCAACTTCGCGGT-3′ and pGp4: 5′-CTGCGTGGTGCTTTACGCGC-3′, respectively.

For the First round, the 30 µl reaction volume contained 3 µl DNA, 25 pmol/µl of each primer (pGp1 and pGp2), 3 µl of 10× PCR buffer (containing 1.5 mM magnesium chloride), 6.0 µl Q-solution, 0.2 mM deoxynucleotide triphosphates (dNTPs) and 1.0 U HotStar *Taq* polymerase (QIAGEN).

For the second run, 1 µl of the first run product was added to 24 µl reaction volume containing, 25 pmol/µl of each primer (pGp3 and pGp4), 2.5 µl of 10× PCR buffer, 5.0 µl Q-solution, 0.2 mM dNTPs and 1.0 U HotStar *Taq* polymerase. Amplification for both rounds for 40 and 35 cycles respectively was carried out in an Eppendorf mastercycler thermal cycler as follows: denaturation at 95°C for 15 min, 94°C for 30 sec, 64°C for 1 min, 72°C for 1 min, 30 sec and a final extension at 72°C for 10 min.

The second round PCR products were electrophoresed in a 2% TAE (0.04 M Tris-acetate and 0.001 M EDTA pH 8.0) agarose gel with ethidium bromide. The size of amplicons was estimated by comparison with 100 bp plus DNA ladder (Fermentas Life Sciences, EU) and visualized using Kodak Gel logic 100 Molecular Imaging System.

### LAMP

The LAMP assay was performed using a set of 6 primers comprising 2 outer primers (Buruli- F3: CGAGAACAGCCTGCACTG, and Buruli- B3: CGGTTGGCGGTCAAAGC).

Two inner primers (Buruli-FIP:GTGCGCCGTGTCCGGTATGGATACGCGATGTCACCTTC and Buruli- BIP: AGGTCCTAGCAACGCTACGCAAATCCGGCAGGCTTCGG), 2 loop primers Buruli-LF: GCCTTTGACGGTCTTCGTC, and Buruli- LB: (CACCGCGATCAATCTGCAC). The primers were designed using Primer Explorer (version 4; EikenChemical, Tokyo, Japan; http://primerexplorer.jp/elamp4.0.0/index.html).

Pocket warmer LAMP (pwLAMP) was performed using a loopamp DNA amplification kit (Eiken Chemical) described previously [32]. Each 25 µl reaction mixture contained 1.6 µM each of FIP and BIP, 0.2 µM each of F3 and B3, 0.8 µM each of LF and LB, 2× reaction mixture (12.5 µl), 1 µl of *Bst* DNA polymerase, 1 µl of fluorescence detection reagent (Eiken Chemical), 3.5 µl distilled water and 1 µl sample.

Reaction tubes were incubated at 60°C for 60 min in the heat block (GeneAmp 9700, Applied Biosystems, Foster City, CA) while with the pwLAMP, the tubes were sandwiched in a twofold pocket warmer (Hokaron Haru-type, Lotte Health Products, Tokyo, Japan) surrounded by a paper towel, and put in a Styrofoam box for 120 min (60 min reaction incubation). The reaction was terminated at 85°C for 5 min and the results were read by eye in ambient light and also using UV illumination.

To evaluate the specificity of the LAMP method for *M. ulcerans*, DNA extracts of eight Mycobacterium sp. (*Mycobacterium marinum*, *Mycobacterium tuberculosis*, *Mycobacterium avium*, *Mycobacterium intracellulare*, *Mycobacterium kansasii*, *Mycobacterium abscessus*, *Mycobacterium chelonae*), two *Mycobacterium ulcerans* strains (*Mycobacterium shinsuence* (Japanese strain) and one (African strain)) and Jurkat (human T cell line) were examined.

### Statistical analysis

A chi-squared test was performed to reveal the statistical difference using SPSS (version 16.0; SPSS Inc., Chicago, IL) software.

## Results

### Detection limit of LAMP for *M. ulcerans*

LAMP reaction requires a constant temperature of about 60°–65°C for 60 min for amplification of DNA [27]–[34]. In a previous study a pocket warmer reached 58°C in 30 min and stayed around 60°C for more than 60 min in a Styrofoam box [34]. The 3 pocket warmers (of a pack of 30 hand warmers) tested in this study achieved a temperature of 60°C after 60 min and maintained this temperature for about 90 min. The pocket warmer thus provided a suitable temperature (60°C) and time range (60 min) for amplification. Both pwLAMP and the conventional LAMP assays were able to detect to the limit of 300 copies of the target sequence after 60 min of amplification. This limit improved to 30 copies when the conventional LAMP was carried out at 65°C (the pocket warmer was not able to attain this temperature and was therefore not investigated).

### Specificity of pwLAMP for *M. ulcerans*

**Figure 1:**
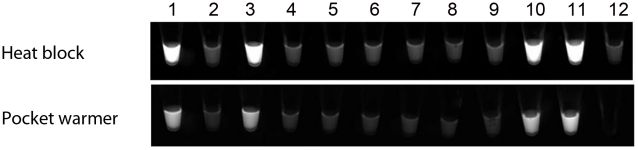
Specificity of Loop mediated isothermal amplification (LAMP) for *Mycobacterium ulcerans*. Conventional (upper, heat block) and pw-LAMP (lower, pocket warmer). Fluorescence image under the UV light are shown. Lanes; 1; *Mycobacterium ulcerans*, 2; *Mycobacterium marinum*, 3; *Mycobacterium shinsuense*, 4; *Mycobacterium tuberculosis*, 5; *Mycobacterium avium*, 6; *Mycobacterium intracellularie*, 7; *Mycobacterium kansasii*, 8; *Mycobacterium abscessus*, 9; *Mycobacterium chelonae*, 10; *Mycobacterium ulcerans*, 11; *Mycobacterium ulcerans* and 12; Jurkart cell line.

Observation under ambient as well as UV illumination demonstrated clearly that the LAMP reaction produced positive signal specifically in DNA from *M. ulcerans*, but not in DNA extracts of *M. marinum*, *M. tuberculosis*, *M. avium*, *M. intracellularie*, *M. kansasii*, *M. abscessus*, *M. chelonae*, and Jurkart, a human T cell line (Figure 1).

### Comparison of LAMP with IS*2404* PCR

**Figure 2:**
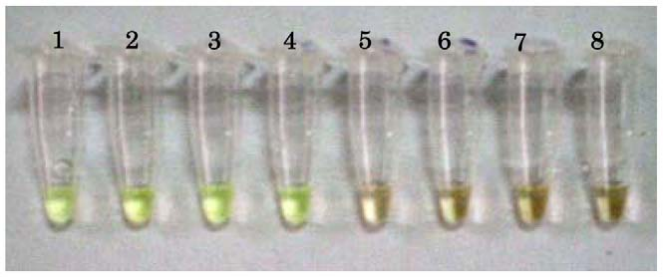
Detection of *Mycobacterium ulcerans* under ambient illumination. Tubes containing *M. ulcerans* DNA produced greenish fluorescence (tubes 1–4).

The sensitivity and specificity of the LAMP assays for the detection of *M. ulcerans* is shown in tables 1 and 2. Under ambient illumination, positive specimens in the LAMP assay produced greenish colouration (Figure 2). When purified DNA extracts were used, 21 (16 swabs, 5 fine needle aspirates) (70%) of 30 clinical specimens were positive by IS*2404* PCR as well as by the conventional LAMP. None of the PCR positive specimens were negative by conventional LAMP. However 19 samples of purified DNA extracts were positive with the pwLAMP, but the 90.5% sensitivity (19/21) of the pwLAMP compared to the results by IS*2404* PCR was not statistically significant (p = 0.58, Chi-square test).

**Table 1:** Comparison of IS*2404* PCR with pocket warmer LAMP for the detection of *Mycobacterium ulcerans* using different DNA templates.

	Pocket warmer LAMP								
	Unboiled extract			Boiled extract			Purified extract		
	(+)	(−)	Total	(+)	(−)	Total	(+)	(−)	Total
IS*2404* PCR (+)	12	9	21	9	12	21	19	2	21
IS*2404* PCR (−)	0	9	9	0	9	9	0	9	9
Total	12	18	30	9	21	30	19	11	30
Positivity	40%			30%			63.3%		
*Sensitivity	100%			100%			90.5%		

*: Sensitivity as compared with IS*2404* PCR.

LAMP (Loop mediated isothermal amplification).

**Table 2:** Comparison of IS*2404* PCR with conventional LAMP for the detection of *Mycobacterium ulcerans* using different DNA templates.

	Conventional LAMP								
	Unboiled extract			Boiled extract			Purified extract		
	(+)	(−)	Total	(+)	(−)	Total	(+)	(−)	Total
IS*2404* PCR (+)	12	9	21	9	12	21	21	0	21
IS*2404* PCR (−)	0	9	9	0	9	9	0	9	9
Total	12	18	30	9	21	30	21	9	30
Positivity	40%			30%			70%		
*Sensitivity	100%			100%			100%		

*: Sensitivity as compared with IS*2404* PCR

LAMP (Loop mediated isothermal amplification).

All negative specimens in IS*2404* PCR were negative in both LAMP assays, indicating specificities of both LAMP assays to the reference method were 100%. Twelve unboiled (9 swabs and 3 fine needle aspirates) and 9 boiled (6 swabs and 3 fine needle aspirates) extracts were positive by all 3 detection assays with sensitivities of 57.1% (unboiled, 12/21) and 42.9% (boiled, 9/21) compared to results using purified DNA extracts respectively for both LAMP and IS*2404* PCR assays. The positivity of swabs was found to be in the range of 30% to 80% compared to 30% to 50% for fine needle aspirates.

When the positivities in crude DNA specimens were compared with those in purified DNA, the differences were statistically significant by chi-square test (unboiled vs purified DNA, p = 0.0195, and boiled vs purified DNA, p = 0.0019). None of the IS*2404* PCR negatives was positive in the LAMP assays irrespective of the DNA extracts type used. These data suggest that sensitivity of LAMP and PCR assays for the detection of *M. ulcerans* in clinical specimens is enhanced when purified DNA extracts are used.

## Discussion

BU is a neglected tropical disease that mostly affects the poor in resource limited communities in sub-Saharan Africa [1]–[3]. IS*2404*PCR, the method of choice for confirmation of BU diagnosis cannot be operational in BU endemic areas [16],[17],[24]–[25].

The development of rapid and reliable point of care diagnostic assays is of high priority to BU management and prevention.

This study explored the potential use of the LAMP method for field diagnosis of BU. Some important limitations to the use of this assay in the field include specimen purification and difficulty in maintaining isothermal condition for the reaction. A study suggested that omission of DNA extraction or the use of crude DNA extracts have no effect on the LAMP test [32]. Hatano *et al* used a disposable pocket warmer to provide isothermal condition for LAMP reaction [34].

Based on this knowledge, we applied the pwLAMP to crude and purified DNA extracts in order to determine whether this method will be suitable for use as a point of care diagnostic test for BU.

Although the pocket warmers used in this study reached 60°C after 1 hr (instead of 30 min in previous studies [34]), both devices achieved the requisite temperature and holding time for executing LAMP reaction, a major advantage in the use of amplification based assay for the detection of an infectious agent under field condition. The pwLAMP did not cross-react with other mycobacteria (Figure 1). Moreover, the experiment on clinical specimens demonstrated that the pwLAMP had a 100% specificity in clinical specimens of BU.

The pwLAMP was found to have comparable sensitivity as the conventional LAMP at 60°C as both assays were able to detect 300 copies of IS*2404* element (equivalent of 1.5 genomes of *M. ulcerans*). The detection limit of the conventional LAMP at 65°C improved to 30 copies of IS*2404* and this level of sensitivity may probably be achieved with a pocket warmer that can attain a temperature of 65°C and maintain a holding time of 60 min. When applied to purified DNA extracts of clinical specimens, the pwLAMP, conventional LAMP and IS*2404* PCR yielded concordant results (Tables 1 and 2).

However, 2 of the samples that were positive by IS*2404* PCR/conventional LAMP were negative by the pw LAMP. None of the IS*2404* PCR negative samples were positive in both types of LAMP assays. On the other hand we observed a drop in detection rate from 63–70% to 30–40% when crude extracts of clinical specimens were used (Tables 1 and 2).

This indicates that the use of crude DNA extracts as template may not be appropriate for the detection of *M. ulcerans* by the LAMP method. This observation contradicts a previous study that suggested omission of DNA extraction had no effect on sensitivity of the LAMP assay [32].

For the crude preparations however, it is noteworthy that the detection rate of the LAMP assay was significantly higher for the unboiled extracts than for the boiled extracts. Explanations for these results were not explored. The observation that the LAMP assay was not inhibited especially for the unboiled specimens is quite consistent with previous work that have shown LAMP to be tolerant to culture medium and to certain biological substances including phosphate buffered saline, serum, plasma, urine and vitreous [32].

In conclusion, the study demonstrates that the LAMP assay yields comparable results as IS*2404* PCR when it is performed at 60°–65°C for 60 min on purified DNA extracts and further supports the use of the pocket warmer as a device for providing isothermal amplification condition for the LAMP assay. This therefore is a potential boost to the application of pwLAMP in resource poor settings.

Challenges of obtaining pure DNA extracts of clinical specimen as well as the use of a pocket warmer capable of maintaining 65°C for 1 hr, however needs to be addressed in order to improve the performance of the pwLAMP assay.

Further development and testing in larger numbers of specimens is therefore necessary to access the potential use of pwLAMP as a simple and rapid point of care diagnostic test for BU.
